# Clinical Evaluation of the Diagnostic Role of MicroRNA-155 in Breast Cancer

**DOI:** 10.1155/2020/9514831

**Published:** 2020-09-07

**Authors:** Fatemeh Hosseini Mojahed, Amir Hossein Aalami, Vahid Pouresmaeil, Amir Amirabadi, Mahdi Qasemi Rad, Amirhossein Sahebkar

**Affiliations:** ^1^Department of Medical Sciences, Mashhad Medical Sciences Branch, Islamic Azad University, Mashhad, Iran; ^2^Department of Biology, Mashhad Branch, Islamic Azad University, Mashhad, Iran; ^3^Department of Biochemistry, Mashhad Medical Sciences Branch, Islamic Azad University, Mashhad, Iran; ^4^Solid Tumors Research Center, Mashhad University of Medical Sciences, Mashhad, Iran; ^5^Reza Radiation Oncology Center, Mashhad, Iran; ^6^Halal Research Center of IRI, FDA, Tehran, Iran; ^7^Biotechnology Research Center, Pharmaceutical Technology Institute, Mashhad University of Medical Sciences, Mashhad, Iran; ^8^Neurogenic Inflammation Research Center, Mashhad University of Medical Sciences, Mashhad, Iran

## Abstract

**Aim:**

Biochemical markers, including microRNAs (miRs), may facilitate the diagnosis and prognosis of breast cancer. This study was aimed at assessing serum miR-155 expression in patients with breast cancer and receptors.

**Methods:**

This case-control study was conducted on 36 patients with breast cancer and 36 healthy individuals. After RNA extraction from the patient's serum, cDNA was synthesized. The expression of miR-155 was measured using RT-qPCR. Demographic and histochemical data were extracted from patient documents. Data were analyzed using the Statistical Package for the Social Sciences (SPSS) software.

**Results:**

The mean age of subjects in breast cancer and control groups was 47.64 ± 8.19 and 47.36 ± 7.52 years, respectively. The serum miR-155 expression was higher in the cancer group (1.68 ± 0.66) compared to the control group (*p* < 0.0001). There was a significant relationship between serum miR-155 expression and the tumor grade (*p* < 0.001), tumor stage (*p* < 0.001), and tumor size (*p* < 0.001) of the patients. However, no relationship between miR-155 expression and the presence of lymph node involvement (*p* = 0.15), HER2 (*p* = 0.79), Ki-67 (*p* = 0.9), progesterone receptor (*p* = 0.54), and estrogen receptors (*p* = 0.84) was found. The ROC curve analysis showed that the AUC was 0.89 (77.78% sensitivity and 88.89% specificity), and the cutoff was 1.4 (Youden index: 0.6667) for detecting breast cancer.

**Conclusion:**

The findings of this study revealed that serum miR-155 may serve as a potential noninvasive molecular biomarker for breast cancer diagnosis and can help predict the grade of the disease.

## 1. Introduction

Breast cancer (BC) is the most prevalent cancer worldwide and also accounts for 22.8% of female cancers [1]. Breast cancer mortality was estimated to be 626679 in 2018 [2]. In Iran, breast cancer accounts for 76% of female cancers, with 8500 new cases each year [3]. The most important risk factors for breast cancer include female gender, age (30 years old and older) [4], positive family history for breast cancer [4], and familial genetic mutations, including mutations in the breast cancer A1 (BRCA1) and BRCA2 genes [5]. Furthermore, women with a history of breast cancer are more likely (20-25%) to develop microscopic cancer in the opposite breast [6]. A positive history for cancer in the endometrium, ovaries, or colon, as well as radiation therapy for Hodgkin's lymphoma, was shown to increase the risk of breast cancer [6]. The gold standard for diagnosis of breast cancer is histopathology [6]. Several tumor markers have been suggested for the evaluation and management of breast cancer including estrogen and progesterone receptors (ER/PR), which are used for the assessment of susceptibility to hormone treatment, and human epidermal growth factor receptor 2 (HER2), which is used to assess the susceptibility to trastuzumab treatment [7].

Microribonucleic Acids (microRNAs) are a large subgroup of noncoding RNAs made up of 18-25 nucleotides [8]. MicroRNAs (miRs) regulate gene expression after transcription. The increased expression of some miRs, including miR-194 and miR-425, was shown in invading breast cancer cells [9]. One of the goals of this study is to investigate the role of miR-155 in women with breast cancer, but its primary goal is to find a biomarker for breast cancer diagnosis based on hormonal receptors. For the first time in this study, we investigated the role of miR-155 in contraceptive drugs and the number of pregnancies. We found a significant difference between the menarche age with the Ki-67 receptor and the tumor stage with contraceptive medication. It is also the first time to provide a diagnostic value of the tumor grade based on the receiver operating characteristic (ROC) curve as well as the Youden index in breast cancer patients.

## 2. Material and Methods

This case-control study was performed on 36 women with breast cancer (BC) who were referred to our Radiotherapy and Oncology Center from March 2017 to March 2018. The Medical Ethics code of the approved study protocol is IR.IAU.MSHD.REC.1396.83. Each subject, regardless of the allocated group, signed a written informed consent form before participation.

### 2.1. Study Population

All patients with the documented diagnosis of breast cancer based on physical examination and imaging and laboratory assessments were included in the case group. Inclusion criteria for subjects in the case group included a documented diagnosis of breast cancer based on histopathology, age between 20 and 60 years old, and the possibility of obtaining blood samples from the subject. Any subject in the case group whose documentation for histopathological diagnosis of breast cancer was absent was excluded from the study. The control group subjects were randomly selected from healthy women who visited the center for a checkup. The inclusion criteria for control subjects were the absence of documented cancer and age between 20 and 60 years old. Exclusion criteria for the control group included a history of polycystic ovary syndrome and a history of any cancer in first-degree relatives.

The clinical data included grading of BC assessed by the Nottingham Grading System: well-differentiated (WD) tumor: grade I, moderately differentiated (MD) tumor: grade II, and poorly differentiated (PD) tumor: grade III. The well-differentiated tumor represented high homology to the normal terminal duct lobular unit, tubule formation (>75%), mild degree of nuclear pleomorphism, and low mitotic count. A moderately differentiated tumor (grade II) is characterized by tubule formation between 10% and 75%. A poorly differentiated tumor is characterized by a marked degree of cellular pleomorphism and frequent mitoses and no tubule formation (<10%) [10]. The type of involvement and stage of cancer, as well as the presence of HER2, PR, and ER, are detected in breast cancer cell biopsy.

### 2.2. Serum Preparation and RNA Extraction

A 5 ml blood sample was incubated at room temperature for 30 minutes and centrifugated at 4000 rpm for 10 minutes. In order to assess total RNA, 200 *μ*l of the serum was extracted. Total RNA was extracted using the Norgen Biotek Plasma/Serum RNA Purification Mini RNA Kit (Ontario, Canada) Cat: 55000 according to the manufacturer's instruction with modification.

### 2.3. cDNA Synthesis and qRT-PCR

Reverse transcription was performed using the BON-miR miRNA 1st-Strand cDNA Synthesis Kit (Bonyakhteh, Iran) cat: BON209001 based on the manufacturer's instructions. The cDNA was synthesized using the thermocycler device for 10 minutes at 25°C, 60 minutes at 42°C, and 10 minutes at 70°C. The qRT-PCR was performed using the BON-miR QPCR (Bonyakhteh, Iran) cat: BON209002 kit. Primarily, 0.5 *μ*l of forward primer (miR-155 or SNORD47), 0.5 *μ*l of universal reverse primer, 6.5 *μ*l of SYBR master mix, and 1 *μ*l of the synthesized cDNA were mixed in the Eppendorf tube. The mixture was placed in the CFX96 Real-Time PCR Detection System (Bio-Rad) with the following temperature program: 1 cycle of 2 minutes at 95°C in the holding stage and 40 cycles of 5 seconds at 95°C and 30 seconds at 60°C in the cycling stage. The sequences of the forward and reverse primers are shown in Table 1.

### 2.4. Assessment of the Quantity of miR-155

The SNORD47 was used for the normalization based on the (Livak) 2^-*ΔΔ*CT^ method. A pooled healthy sample was prepared by mixing the vortexed samples of 36 healthy individuals. The pooled sample was used for a calibrator. *C*_t_ was assessed for each sample based on the previously mentioned method, and the difference between *C*_t_ of the sample and pooled sample *C*_t_ (Δ*C*_t_) was calculated as follows:

(1) $$\begin{array}{c} \Delta C_{\text{t}}=C_{\text{t}}\;\left(\text{miR}‐155\right)-{\text{C}}_{\text{t}}\;\left(\text{SNORD}47\right). \end{array}$$

Then, ΔΔ*C*_t_ and the normalized value for each sample were calculated as follows:

(2) $$\begin{array}{c} \Delta \Delta C_{\text{t}}=\Delta C_{\text{t}}\;\left(\text{patient or control sample}\right)-\Delta C_{\text{t}}\;\left(\text{calibrator}\right). \end{array}$$

### 2.5. Statistical Analysis

Data were assessed using the Statistical Package for the Social Sciences (SPSS) software (IBM Inc., Chicago, IL, USA) version 20. Graphs were created using GraphPad Prism 8.0 (GraphPad Software Inc., California). Data were checked for normality using the Kolmogorov-Smirnov test. Mean or median and standard deviation (SD) were used to present continuous variables, while frequency and percentage were used to present categorical variables. Comparison between groups was performed using the Student *t*-test and one-way and two-way analysis of variance (ANOVA). A correlation between study parameters was assessed using the Pearson correlation coefficient. The receiver operating characteristic (ROC) curve and the area under the curve (AUC) were performed to assess the diagnostic value of miR-155 for the detection of BC and differentiation between grades, stages, lymph node metastasis, tumor size (T size), HER2, ER, PR, and Ki-67. The cutoff value for miR-155 for each diagnosis was calculated using the Youden index. Binary logistic regression and linear regression were performed to assess the relationship between study parameters and BC. The *p* value lesser than 0.05 was considered statistically significant.

## 3. Results

A total of 72 subjects (36 BC patients and 36 controls) participated in this study. The mean age of the subjects in BC and control groups was 47.64 ± 8.18 and 47.36 ± 7.52 years, respectively. The mean of the body mass index (BMI) in the breast cancer patients and control group was 27.70 ± 4.62 and 26.35 ± 3.94 kg/m^2^, respectively. The mean number of pregnancies in the BC patients and control group was 3.22 ± 1.94 and 3.33 ± 1.95, respectively (Table 2).

Demographic characteristics of study subjects are presented in Tables 2 and 3 . There were no significant difference between the breast cancer and control groups in terms of age (*p* = 0.881), BMI (*p* = 0.186), number of pregnancies (*p* = 0.810), age of menarche (*p* = 0.306), history of abortion (*p* = 0.635), and contraceptive drug usage (*p* = 0.475).

Clinical characteristics of the breast cancer group are shown in Table 4. The most common tumor grade was MD (15, 41.7%), followed by PD (11, 30.6%). The most common cancer stage was stage II (17, 47.2%), followed by stage III (11, 30.6%). The most common types of receptors were HER2 negative (27, 75%), PR positive (19, 52.8%), ER positive (24, 66.7%), and Ki‐67 > 10% (22, 61.1%).

**Figure 1:**
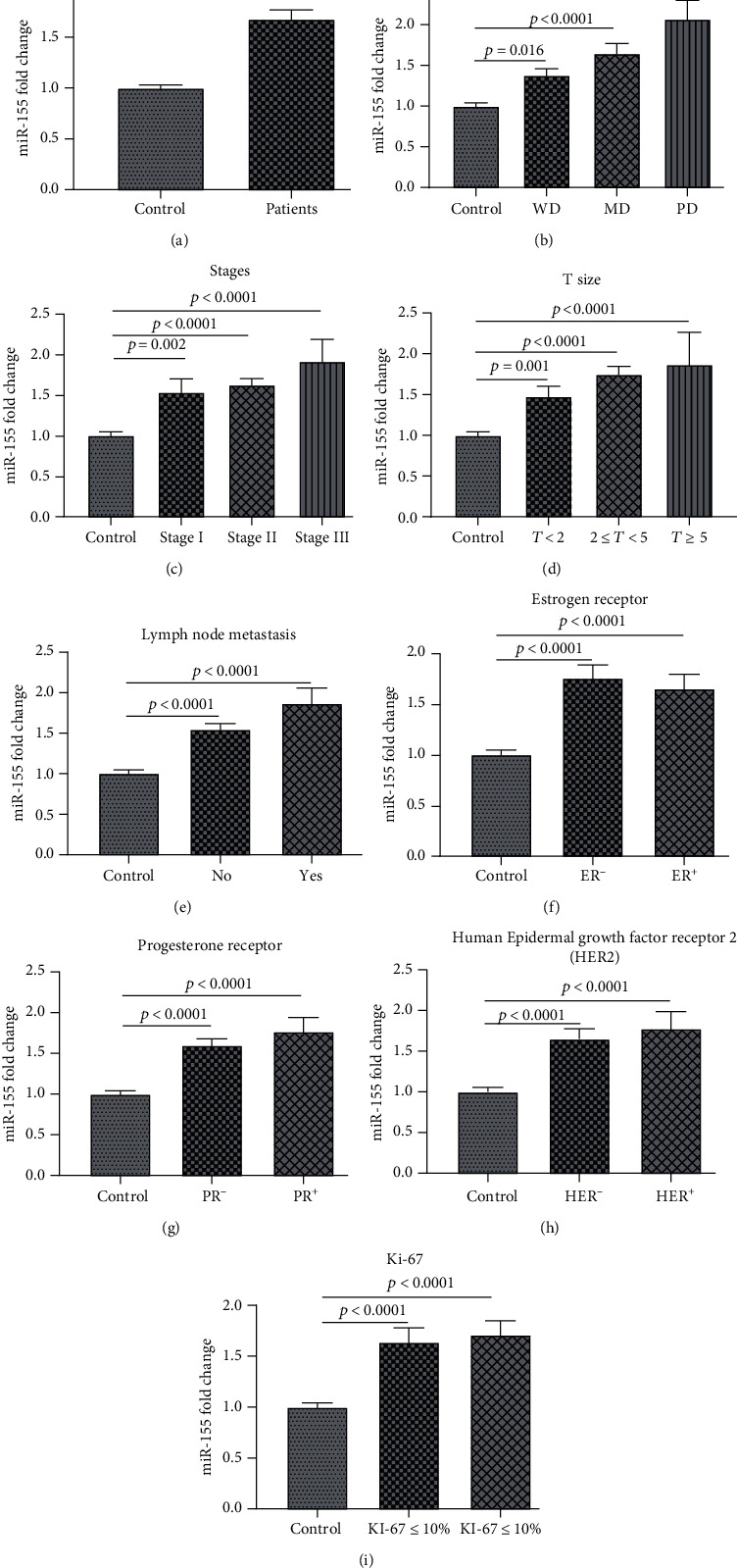
The (a) *t*-test and (b–i) ANOVA comparison between *x*-fold expression of miR-155 in the BC subgroups and control group.

The comparison of miR-155 expression between study groups is presented in Table 4. The expression of miR-155 in BC patients was 1.68 ± 0.66 times greater than that in the control group (*p* < 0.0001). The miR-155 expression was significantly higher in all grades, stages and T sizes, and lymph node metastases as well as ER, PR, Ki-67, and HER2 categories compared to that of the control group (*p* < 0.001) (Table 4 and Figure 1).

The miR-155 expression was significantly higher in WD, MD, and PD grades compared to that of controls (*p* = 0.016, *p* < 0.001, and *p* < 0.001, respectively). The miR-155 expression was significantly higher in grade III than in grade I (*p* = 0.011) (Table 4 and Figure 1).

The expression of miR-155 was significantly higher in stage I, stage II, and stage III compared to that of the control group (*p* = 0.002, *p* < 0.001, and *p* < 0.001, respectively). However, there was not any significant difference between miR-155 in stage II compared to stages I and III. The multivariate analysis with BMI as a confounder revealed a considerable difference in terms of miR-155 expression and stage (*p* = 0.034) (Table 4 and Figure 1).

The expression of miR-155 was significantly higher in the large tumor size (T size) compared to that of the control group (*p* < 0.001). The expression of miR-155 was significantly higher in T1, T2, and T3 compared to that of the control group (*p* = 0.001, *p* < 0.001, and *p* < 0.001, respectively), and also, there was a significant difference between T1, T2, and T3 in the patient group (*p* < 0.001) (Table 4 and Figure 1).

The expression of miR-155 was significantly greater in lymph node involvement compared to that of the control group (*p* < 0.001). The miR-155 expression was higher in positive and negative lymph node metastases compared to that of the control group (*p* < 0.001 each group), but there was no significant difference between positive and negative lymph node involvement (*p* = 0.15) (Table 4 and Figure 1).

The expression of miR-155 was significantly higher in the estrogen receptor (ER) compared to that of the control group (*p* < 0.001). The expression of miR-155 was significantly higher in ER^+^ and ER^−^ compared to that of the control group (*p* < 0.001 each group). However, there was not any significant difference between miR-155 in ER^+^ compared to ER^−^ (*p* = 0.84) (Table 4 and Figure 1).

The expression of miR-155 was significantly higher in the progesterone receptor (PR) compared to that of the control group (*p* < 0.001). The expression of miR-155 was significantly higher in PR^+^ and PR^−^ compared to that of the control group (*p* < 0.001 each). However, there was not any significant difference between miR-155 in PR^+^ compared to PR^−^ (*p* = 0.54) (Table 4 and Figure 1).

The expression of miR-155 was significantly higher in HER2 compared to that of the control group (*p* < 0.001). The expression of miR-155 was significantly higher in HER^+^ and HER^−^ compared to that of the control group (*p* < 0.001). However, there was not any significant difference between miR-155 in HER^+^ compared to HER^−^ (*p* = 0.79) (Table 4 and Figure 1).

The expression of miR-155 was significantly higher in Ki-67 compared to that of the control group (*p* < 0.001). The expression of miR-155 was significantly higher in Ki‐67 ≤ 10% and Ki‐67 > 10% compared to that of the control group (*p* < 0.001 each). However, there was not any significant difference between miR-155 in Ki‐67 ≤ 10% compared to Ki‐67 > 10% (*p* = 0.9) (Table 4 and Figure 1).

**Figure 2:**
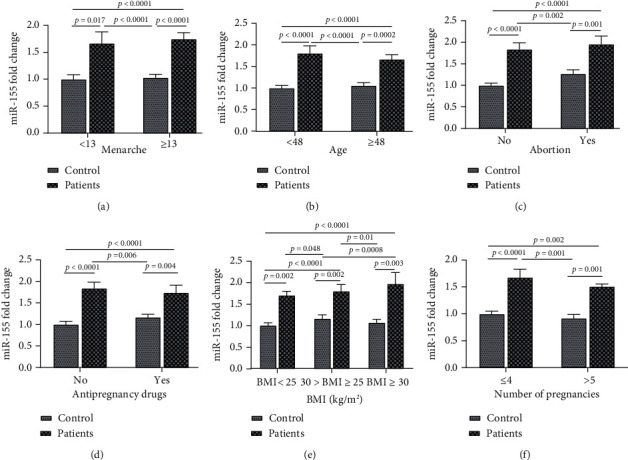
The Tukey multiple comparisons of the serum expression level of miR-155 among study groups. Comparison of serum expression of miR-155 between (a) menarche age groups, (b) age groups, (c) abortion categories, (d) contraceptive drugs, (e) BMI groups, and (f) number of pregnancies.

Two-way ANOVA results showed that age, BMI, number of pregnancies, age of menarche, contraceptive drug usage, and history of abortion had no significant effect on expression level (*p* > 0.05), and the difference between groups was due to BC for age, BMI, number of pregnancies, antipregnancy drugs, and abortion (*p* < 0.0001) (Table 5 and Figure 2).

The relationship between miR-155 expression and the tumor grade, tumor stage, T size, node metastasis, and tumor markers is presented in Tables 6 and 7. The binary logistic regression revealed that miR-155 expression and the grade and stage of the tumor were the predictors of BC (*p* < 0.001, *p* = 0.03, and *p* = 0.048), respectively. It was shown that probability of BC increased by 6.15 times for every one-unit increase in mir-155 expression. Similarly, a one-unit increment in the grade and stage of the tumor was associated with 10.28 and 7.61 times increased risk of BC. Also, this analysis was performed for each parameter such as tumor grade and stage, T size, node metastasis, ER, PR, HER2, and Ki-67, compared to age, BMI, number of pregnancies, contraceptive drug usage, history of abortion, and age of menarche (Tables 6 and 7).

**Figure 3:**
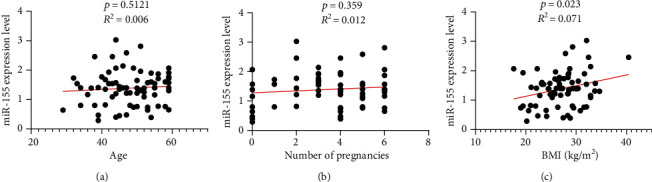
The linear regression analysis between miR-155 expression and age, number of pregnancies, and BMI.

The linear regression analysis was performed between miR-155 and age, BMI, and number of pregnancies. In this study, it was found that miR-155 had no relationship with age and number of pregnancies (*p* = 0.51 and *p* = 0.35, respectively), while there was a significant relationship with BMI (*p* = 0.023) (Figure 3).

**Figure 4:**
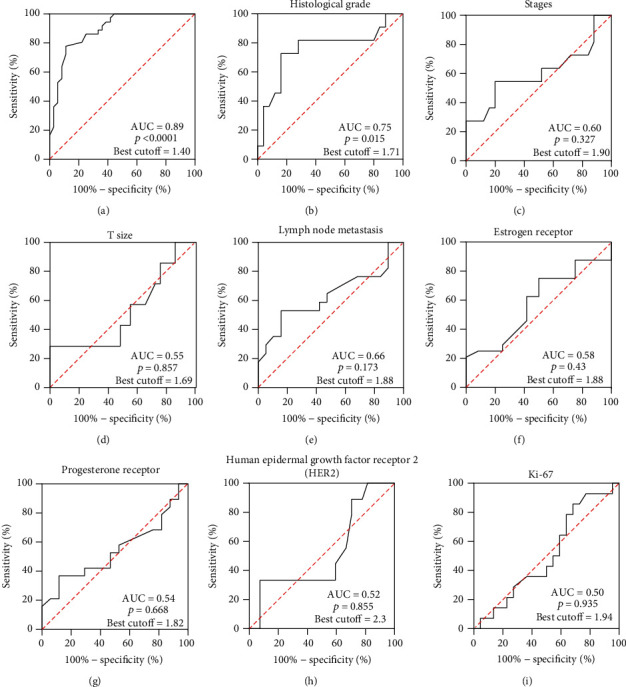
The ROC curve for miR-155 in the detection of BC and differentiation of pathological categories.

The ROC curve was used to identify the sensitivity and specificity of the tumor grade (*p* = 0.015), tumor stage (*p* = 0.328), T size (*p* = 0.857), node metastases (*p* = 0.173), PR (*p* = 0.669), ER (*p* = 0.430), HER2 (*p* = 0.855), and Ki-67 (*p* = 0.935) (Tables 8 and 9 and Figure 4).

Based on the ROC curve, the optimal cutoff in the expression of miR-155 for detecting BC was 1.40 (Youden index: 0.6667), which resulted in the sensitivity and specificity of 77.78% (95% CI: 61.92% to 88.28%) and 88.89% (95% CI: 74.69% to 95.59%), respectively (Table 8).

**Figure 5:**
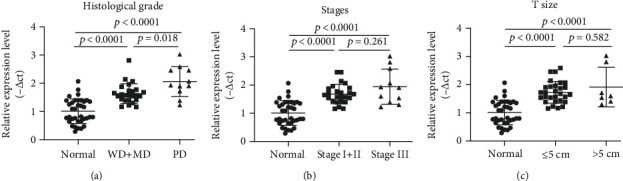
The Youden index three-group model for the role of miR-155 in the detection and differentiation of tumor grades, tumor stages, and tumor size in BC patients and comparison with healthy subjects: (a) relative expression level of miR-155 for low-grade (WD+MD) compared to high-grade (PD) and healthy groups, (b) relative expression levels of miR-155 for low-stage (I+II) compared to high-stage (III) and healthy groups, and (c) comparison of relative expression levels of miR-155 between ≤5 cm and > 5 cm tumor sizes and healthy group.

If the miR-155 expression was used as the biomarker for BC grades, at the Youden cutoff of 0.3626, it could identify low-grade (WD and MD) from high-grade (PD) BC with a sensitivity of 99.76% and specificity of 88.25% (Table 9 and Figure 5).

If the estrogen receptor (ER) was used as a biomarker for distinguishing BC in patients, expression of miR-155 at the Youden cutoff of 0.3397 could identify the healthy group from ER^−^ and ER^+^ in BC with a sensitivity of 80.75% and specificity of 80.64% (Table 9).

## 4. Discussion

Although histochemistry is considered the standard method for the diagnosis of breast cancer, it still faces challenges in the preanalytical and analytical stages of cancer [11]. The preanalytical factors that may influence the histochemistry methods include delay in tissue fixation, type of chemical fixation, and duration of tissue fixation. At the same time, the analytical stage might be affected by the detection of cutoff and personal variations in the visual examination of the tissue samples [12]. Based on the challenges in the histochemistry methods, it was suggested to add biochemical parameters to assist the diagnosis of breast cancer [9].

The findings of this study revealed that the mean age of the subjects with breast cancer was 47.36 years which was similar to the finding previously reported in Iran [13] but lower than those in the previously reported studies conducted in other countries [14, 15]. The findings of this study, along with the results of the previous studies, indicate that the incidence of breast cancer in Iran is higher in younger ages than that in Western countries.

In this study, the expression of miR-155 in BC patients was 1.68 ± 0.66 times more than that in the control group. This finding was in line with the results of the previous studies that reported increased miR-155 expression by 2.62 to 8.8-fold in breast cancer patients compared to controls [16]. In the studies of Guo et al. [15], Sun et al. [17], and Zhang et al. [18], the increase in miR-155 expression in BC was 2.94 times, 2.62 times, and 2.87 times, respectively.

The findings of this study revealed that the highest expression of miR-155 was among grade 3 (PD) of breast cancer. Furthermore, the miR-155 expression in WD, MD, and PD was 1.38 ± 0.3- (*p* = 0.016), 1.67 ± 0.52- (*p* < 0.001), and 2.07 ± 0.81- (*p* < 0.001) fold higher than that of the healthy controls. This finding was in line with the results of a previous study that reported increased miR-155 expression with an increased tumor grade (*p* = 0.012) [19].

The findings of this study revealed that the miR-155 expression increases with the increased stage of the tumor (*p* < 0.001). This finding was in line with the results of previous studies that reported increased miR-155 expression with an increased tumor stage (*p* = 0.001 and *p* = 0.002 [19, 20]), and also, in previous studies, a significant relationship was observed between miR-155 expression and the stage of breast cancer tumor [15, 21]. However, Mar-Aguilar et al. [13] and Sun et al. [17] reported no significant relationship between miR-155 expression and the stage of breast cancer tumors (*p* > 0.05 and *p* = 0.066, respectively). In contrast to a previous study, the miR-155 expression was highest in stages II and III compared to stages I and IV in only one study [14].

The findings of this study also revealed a significant link between the tumor size and the miR-155 expression (*p* < 0.001). It was in line with the study of Lu et al. [12]. Similar to our research, the most frequent tumors were 20 to 50 mm in size, and miR-155 expression was significantly higher in this size than in other sizes. However, there was no significant relationship between miR-155 expression and the tumor size in the studies of Sun et al. (*p* = 0.066) and Chen et al. (*p* = 0.947) [17, 19].

Increased expression of miR-155 was observed in the BC group, and there was a statistically significant relationship between the expression level and lymph node metastasis. Lymph node involvement was observed in 17 (47.2%) subjects, but there was no significant difference in miR-155 expression between lymph node involvement and noninvolvement in this study (*p* = 0.15). This finding was in line with Sun et al.'s study (*p* = 0.142) [17] while this finding was in contrast with the previously reported relationship between miR-155 expression and lymph node invasion in previous studies which was confirmed by the studies of Chen et al. (*p* = 0.003) [19], Zheng et al. (*p* = 0.034) [20], Elshimy et al. (*p* = 0.05) [22], and Amal Fawzy et al. [23].

In addition to myriad risk factors, most notably age, family history, and hormonal factors, some various behaviors and characteristics can be classified into breast cancer, including the histologic features of the malignant tumor grade, tumor stage, and indices measurable by immunohistochemistry, most commonly PR, ER, HER2, and Ki-67 [21, 24].

The result of the current study revealed no linkage between miR-155 expression and PR (*p* = 0.54), ER (*p* = 0.84), and HER2 (*p* = 0.79) positivity. These results were partly in line with the findings of previous studies [12, 17]. There is controversy regarding the relationship between miR-155 expression and PR positivity. While similar findings were reported regarding the link between miR-155 expression and PR positivity in one study [17], no relationship was observed between miR-155 expression and PR positivity [12]. In this study, 25% of patients were HER2-positive and 75% HER2-negative, and 66.7% were ER-positive and 33.3% ER-negative. There was no significant relationship between miR-155 expression and HER2 (*p* = 0.79) and ER (*p* = 0.84) positivity. The results are corroborated by the studies of Lu et al. [12], Sun et al. (HER2 (*p* = 0.123), ER (*p* = 0.451)) [17], and Chen et al. [19] (ER (*p* =0.977), PR (*p* = 0.09)).

This study also failed to find a significant effect for Ki-67 on fold expression of miR-155 among BC patients (*p* = 0.9). This finding was in line with the previous study on 45 BC patients. Zheng et al. showed that upregulated miR-155 expression was associated with a higher proliferation index (Ki‐67 > 10%) (*p* = 0.019) [20]. However, Bašová et al. [25] reported the link between miR-155 expression and Ki‐67 ≥ 20% in 134 patients (*p* = 0.013).

There was no significant difference in the expression of miR-155 between cancer and control groups in terms of age (*p* = 0.899). There was no any linkage between the expression of miR-155 in BC patients < 48 years old compared to the healthy group with the same age (*p* = 0.925) and also in BC groups ≥ 48 years compared to the healthy group (*p* = 0.873). This result was in line with the finding of Guo et al. [15]. They showed that there is no relationship between miR-155 in the BC group < 45 and ≥45 years old (*p* = 0.67). Chen et al. [19] reported that they did not find any significant difference between miR-155 expression and age groups (*p* = 0.389).

This study revealed a significant effect for abortion on *x*-fold expression of miR-155 in the BC group. In this study, a significant relationship was found between those who had a history of abortion and those who had no history (*p* = 0.045). This result was in line with the findings of previous studies. Guo et al. [15] reported that the history of abortion has a direct effect on upregulated miR-155 expression (*p* = 0.01).

To the best of our information, this study was the first paper that assessed the miR-155 expression in BC patients based on contraceptive drug usage (*p* = 0.557). The miR-155 expression in patients who had the background of using contraceptive drugs and in patients who had never use these drugs was, respectively, 1.74 ± 0.74- (*p* = 0.04) and 1.84 ± 0.67- (*p* < 0.001) fold higher than that in the healthy controls.

To the best of our knowledge, this study was the first study that assessed the miR-155 expression in BC patients based on their number of pregnancies. Although no significant difference in miR-155 fold expression and number of pregnancies (*p* = 0.266), the miR-155 expression in patients who had ≤4 parturitions and in patients who had >5 calving was, respectively, 1.68 ± 0.77- (*p* <0.001) and 1.51 ± 0.23- (*p* <0.001) fold higher than that in the healthy controls.

The study also examined the association between miR-155 and menarche age. There was no significant association between miR-155 in patients under 13 years and over 13 years of menarche age (*p* = 0.741). The expression of miR-155 in patients younger than 13 years was 1.67 times higher than that in healthy subjects < 13 years (*p* = 0.0017). Also, the expression of miR-155 was 1.75 times higher in BC subjects ≥ 13 years old compared to the healthy group with the same age (*p* < 0.001). While this finding was in contrast with the previously reported relationship between miR-155 expression and menarche age, Guo et al. [15] showed that single-factor analysis of miR-155 expression among clinical pathologies indicated that miR-155 expression significantly differed among patients according to menarche age (*p* = 0.004). They also reported that subjects with a menarche age of <13 years, several artificial abortions, high BMI, and a family history of breast cancer had a relatively high miR-155 expression [15].

The current study found that the expression of miR-155 was significantly higher in the cancer group compared to controls in all BMI categories.

In Guo et al.'s study [15], menarche age under 13 and BMI over 24 kg/m^2^ were significantly associated with increased miR-155 expression, whereas in this study, there was only a relationship between BMI and expression level. There was an increase in BMI, although there was no statistically significant relationship between the patient groups (*p* = 0.437). Consistent with the previous study reported by Guo et al., the mean expression of miR-155 compared to that of the healthy group in terms of BMI less than 25 kg/m^2^, between 25 kg/m^2^ and 30 kg/m^2^, and more than 30 kg/m^2^ was 1.7 ± 0.33 (*p* = 0.002), 1.8 ± 0.67 (*p* = 0.0025), and 1.97 ± 0.92 (*p* = 0.0034), respectively (*p* = 0.003) [15].

The ROC curve is a graphical presentation of screening properties to determine the best cutoff point. The AUC, sensitivity, and specificity of miR-155 were found to be 0.89, 77.78%, and 88.89%, respectively (*p* < 0.0001), and the cutoff was 1.4 (Youden index: 0.6667). In a previous study, Mar-Aguilar et al. [13] reported that the AUC for miR-155 for the detection of BC was 0.99 (95% CI: 0.9866 to 1.0022), and the sensitivity and specificity of miR-155 were reported to be 94.40% and 100%, respectively, and the optimal cutoff was 7.92. In another study, the AUC, sensitivity, and specificity of the miR-155 for detecting BC were reported to be 0.879 (95% CI: 0.820-0.868), 84.2%, 88.1%, respectively, and the cutoff value was 1.24 [15]. Han et al. [14] reported that the AUC, sensitivity, and specificity of miR-155 for detecting BC were 0.749, 100%, and 51.02%, respectively, and the cutoff value was -1.17. Zhang et al. [18] showed that the AUC, sensitivity, and specificity of the miR-155 for detecting BC were 0.692 (95% CI: 0.625-0.754), 66.0%, and 68.9%, respectively, and the cutoff value was 0.321 (Youden index: 2.2). In another study, Sun et al. [17] reported that the AUC for miR-155 for the detection of BC was 0.801 (95% CI: 0.734 to 0.868), the sensitivity and specificity of miR-155 were reported to be 65.0% and 81.8%, respectively, and the optimal cutoff was 1.91.

The findings of this study also showed that miR-155 expression could be used in the differentiation of BC grades with a sensitivity of 81.82%, a specificity of 72%, and the cutoff of 1.71 (Youden index: 0.3626) (*p* = 0.015). To the best of our knowledge, no previous study assessed the sensitivity and specificity of miR-155 for differentiating between BC tumor grades.

One of the limitations of this study was the difficulty in obtaining consent from women to participate in the study along with the missing data in patient documents and the high cost of diagnostic kits, which resulted in the restriction of sampling due to the limited budget of the study. The findings of this study justify the need for further studies with a higher budget in the early detection of breast cancer by using biochemical markers.

In summary, the findings of this study indicate that the miR-155 expression can assist in diagnosis, prognosis, and TNM grading, including lymph node involvement and metastasis in breast cancer patients.

## Figures and Tables

**Table 1:** The sequences of forward and reverse designed primers for target genes.

*Gene name*

Primers (5′ → 3′)

*hsa-miR-155-5p* *Forward:* UUAAUGCUAAUCGUGAUAGGGGUU

*SNORD47* *Forward:* CGCCAATGATGTAATGATTCTG

*Universal reverse primer* Universal reverse primers were obtained from Bonyakhteh Company (Bonyakhteh, Tehran, Iran)

**Table 2:** The comparison of age, BMI, and number of pregnancies between control and patient groups.

Groups		*N*	Mean ± SD	*p*
Age	Control	36	47.36 ± 7.52	0.881
	Patients	36	47.64 ± 8.18	

BMI	Control	36	26.35 ± 3.94	0.186
	Patients	36	27.70 ± 4.62	

Number of pregnancies	Control	36	3.33 ± 1.95	0.810
	Patients	36	3.22 ± 1.94	

**Table 3:** The demographic characteristics of study subjects as per study groups.

Groups	Control group frequency (%)	Cancer group frequency (%)	*p*
Menarche			
<13	9 (25)	13 (36.1)	0.306
≥13	27 (75)	23 (61.9)	

Abortion			
Yes	17 (47.2)	15 (41.7)	0.635
No	19 (52.8)	21 (58.3)	

Contraceptive drugs			
Yes	14 (38.9)	17 (47.2)	0.475
No	22 (61.1)	19 (48.7)	

**Table 4:** Comparison of miR-155 expression among clinical categories.

Pathological categories	Sample size	*x*-fold expression ± SD (vs. control)	*p* (ANOVA^ǂ^)	*p* (vs. control) (Tukey^ᵻ^)
Normal	36	1 ± 0.33		
Histology grade				
WD	10	1.38 ± 0.3	<0.001	0.016
MD	15	1.67 ± 0.52		<0.001
PD	11	2.07 ± 0.81		<0.001

TNM stage				
Stage I	8	1.53 ± 0.5	<0.001	0.002
Stage II	17	1.62 ± 0.38		<0.001
Stage III	11	1.91 ± 0.94		<0.001

Tumor size (*T*)				
T1 (*T* < 2)	11	1.48 ± 0.45	<0.001	0.0015
T2 (2 ≤ *T* < 5)	18	1.75 ± 0.47		<0.001
T3 (*T* ≥ 5)	7	1.87 ± 1.08		<0.001

Lymph node involvement (*N*)				
Yes	17	1.86 ± 0.82	0.15	<0.001
No	19	1.54 ± 0.37		<0.001

Estrogen receptor (ER)				
ER+	24	1.65 ± 0.73	0.84	<0.001
ER-	12	1.75 ± 0.49		<0.001

Progesterone receptor (PR)				
PR+	19	1.77 ± 0.81	0.54	<0.001
PR-	17	1.60 ± 0.38		<0.001

HER2				
HER+	9	1.78 ± 0.66	0.79	<0.001
HER-	27	1.65 ± 0.65		<0.001

Ki-67				
≤10%	14	1.64 ± 0.56	0.9	<0.001
>10%	22	1.71 ± 0.71		<0.001

WD = grade 1; MD = grade 2; PD = grade 3; ER = estrogen receptor; PR = progesterone receptor; HER2 = human epidermal growth factor receptor 2. ^ǂ^The analysis of variance (ANOVA) was performed for the analysis. ^ᵻ^Tukey multiple comparison.

**Table 5:** Two-way ANOVA results for age, age of menarche, history of abortion, contraceptive drug usage, and BMI in the patient and control groups.

Groups	Sample size		*x*-fold expression (vs.control groups) ± SD	*p* ^ǂ^	*p* ^¥^ (tumor vs. normal)
	Control group	Cancer group			
Age					
<48 y	18	18	1.81 ± 0.79	Age: 0.899^ǂ^	0.925
≥48 y	18	18	1.67 ± 0.53	BC: <0.0001^ǂ^	0.873

Menarche age					
<13	9	13	1.67 ± 0.79	Menarche: 0.741^ǂ^	0.0017
≥13	27	23	1.75 ± 0.59	BC: <0.0001^ǂ^	<0.001

Abortion					
Yes	17	15	1.96 ± 0.76	Abortion: 0.045^ǂ^	0.001
No	19	21	1.84 ± 0.72	BC: <0.0001^ǂ^	<0.001

Contraceptive drugs					
Yes	14	16	1.74 ± 0.74	Contraceptive drugs: 0.557^ǂ^	0.004
No	22	20	1.84 ± 0.67	BC: <0.0001^ǂ^	<0.001

Number of pregnancies					
≤4	20	22	1.68 ± 0.77	Pregnancy number: 0.266^ǂ^	<0.001
>5	16	14	1.51 ± 0.23	BC: <0.0001^ǂ^	<0.001

BMI					
BMI < 25 kg/m^2^	14	9	1.7 ± 0.33	BMI: 0.437^ǂ^	0.002
30 > BMI ≥ 25 kg/m^2^	16	16	1.8 ± 0.67	BC: <0.0001^ǂ^	0.0025
BMI ≥ 30 kg/m^2^	6	11	1.97 ± 0.92		0.0034

^ǂ^The two-way analysis of variance (ANOVA) was used for the comparison. ^¥^Tukey multiple comparison.

**Table 6:** The binary logistic regression analysis between miR-155 and age, BMI, number of pregnancy, age of menarche, history of abortion, and contraceptive drug usage on subject groups.

Parameters		*p*	OR	95% CI for OR	
				Lower	Upper
Patients vs. control	miR-155	0.0001^∗^	4.115	1.890	6.460
	Age	0.251	0.938	0.847	1.047
	BMI	0.306	1.135	0.885	1.357
	Number of pregnancies	0.508	0.847	0.086	1.642
	Menarche age	0.166	0.341	0.073	1.254
	Abortion	0.196	0.367	0.313	4.813
	Contraceptive drugs	0.616	1.455	0.785	2.426

**Table 7:** The binary logistic regression analysis between BC and study parameters and miR-155, age, BMI, age of menarche, history of abortion, and contraceptive drug usage.

Groups		*p* value	OR	95% CI for OR		Groups		*p* value	OR	95% CI for OR	
				Lower	Upper					Lower	Upper
Grade	miR-155	0.030^∗^	10.283	1.253	84.398	ER	miR-155	0.834	0.839	0.163	4.327
	Age	0.953	0.997	0.896	1.109		Age	0.074	1.099	0.991	1.219
	BMI	0.922	0.991	0.827	1.188		BMI	0.564	0.950	0.798	1.131
	Menarche age	0.764	0.757	0.123	4.666		Menarche age	0.618	1.534	0.285	8.252
	Abortion	0.096	4.383	0.771	24.918		Abortion	0.188	0.333	0.065	1.714
	Contraceptive drugs	0.628	1.542	0.267	8.888		Contraceptive drugs	0.474	1.800	0.360	9.008

TNM stages	miR-155	0.048^∗^	7.612	1.021	56.785	PR	miR-155	0.198	3.178	0.546	18.50
	Age	0.683	1.025	0.910	1.155		Age	0.720	0.984	0.899	1.077
	BMI	0.102	0.847	0.694	1.034		BMI	0.437	0.935	0.789	1.108
	Menarche age	0.478	1.999	0.295	13.562		Menarche age	0.905	1.096	0.244	4.909
	Abortion	0.181	3.403	0.565	20.500		Abortion	0.225	0.400	0.091	1.757
	Contraceptive drugs	0.050^∗^	6.665	0.996	44.590		Contraceptive drugs	0.472	1.721	0.392	7.560

T size	miR-155	0.232	3.426	0.455	25.794	HER2	miR-155	0.532	1.762	0.299	10.39
	Age	0.412	1.054	0.929	1.197		Age	0.406	0.956	0.859	1.063
	BMI	0.728	0.965	0.787	1.182		BMI	0.868	1.016	0.845	1.220
	Menarche age	0.647	0.647	0.101	4.162		Menarche age	0.694	1.421	0.246	8.203
	Abortion	0.619	1.630	0.238	11.172		Abortion	0.653	1.475	0.272	8.003
	Contraceptive drugs	0.155	4.411	0.571	34.084		Contraceptive drugs	0.122	3.964	0.692	22.68

*N*	miR-155	0.130	3.823	0.675	21.664	Ki-67	miR-155	0.847	1.175	0.228	6.041
	Age	0.823	0.989	0.898	1.089		Age	0.999	1.000	0.904	1.106
	BMI	0.649	1.042	0.874	1.242		BMI	0.407	1.083	0.897	1.307
	Menarche age	0.171	3.020	0.620	14.712		Menarche age	0.043^∗^	5.305	1.058	26.60
	Abortion	0.785	1.230	0.279	5.420		Abortion	0.623	0.672	0.138	3.28
	Contraceptive drugs	0.776	1.242	0.279	5.526		Contraceptive drugs	0.659	1.423	0.298	6.79

**Table 8:** Biomarker index of miR-155 for breast cancer identification using the ROC curve.

Parameters	AUC	Sensitivity (95% CI)	Specificity (95% CI)	Best cutoff	*p*
Control/BC	0.89	77.78% (61.92%-88.28%)	88.89% (74.69%-95.59%)	1.40	<0.0001^∗^
Grade	0.75	81.82% (52.30%-96.77%)	72.0% (52.42%-85.72%)	1.71	0.015^∗^
Stage	0.60	54.55% (28.01%-78.73%)	80.0% (60.87%-91.14%)	1.90	0.327
T size	0.55	42.86% (15.82%-74.95%)	51.72% (34.43%-68.61%)	1.69	0.857
LNM (*N*)	0.66	52.94% (30.96%-73.83%)	84.21% (62.43%-94.48%)	1.885	0.173
HER2	0.52	33.33% (12.06%-64.58)	92.59% (76.63%-98.68%)	2.30	0.855
PR	0.54	42.11% (23.14%-63.72%)	70.59% (46.87%-86.72%)	1.82	0.668
ER	0.58	75.0% (55.10%-88.00%)	50.0% (25.38%-74.62%)	1.88	0.43
Ki-67	0.50	85.71% (60.06%-97.46%)	31.82% (16.36%-52.68%)	1.94	0.935

**Table 9:** Biomarker index of miR-155 for identification of breast cancer by the Youden index three-group model.

Parameters	Sensitivity	Specificity	Youden index	95% CI for Youden	Best cutpoints	
					Lower	Upper
Grade	99.76%	88.25%	0.3626	0.27-0.455	1.528	2.638
Stage	42.40%	77.19%	0.4520	0.3055-0.5985	1.335	2.064
Tumor size	35.67%	79.03%	0.4269	0.261-0.5928	1.362	2.172
LNM (*N*)	44.94%	75.17%	0.4588	0.3231-0.5945	1.307	1.974
HER2	38.82%	79.93%	0.3444	0.1939-0.4949	1.376	1.985
PR	35.46%	77.33%	0.4246	0.2921-0.5572	1.337	2.038
ER	80.75%	80.64%	0.3397	0.2332-0.4462	1.387	1.484
Ki-67	28.37%	79.8%	0.3467	0.2156-0.4778	1.363	2.070

## Data Availability

The data used to support the findings of this study are available from the corresponding author upon request.

## Supplementary Materials

Supplementary Materials Graphical abstract.
